# A Mini-Review of Work Stress and Mindfulness: A Neuropsychological Point of View

**DOI:** 10.3389/fpsyg.2022.854204

**Published:** 2022-04-15

**Authors:** Fátima González-Palau, Leonardo Adrián Medrano

**Affiliations:** ^1^Instituto de Organizaciones Saludables, Universidad Siglo 21, Córdoba, Argentina; ^2^Neurorehabilitation Unit, Neurology Service, Sanatorio Allende Cerro Hospital, Córdoba, Argentina; ^3^Vicerrectoria de Investigación, Pontifica Universidad Católica Madre y Maestra, Santiago de los Caballeros, Dominican Republic

**Keywords:** stress, mindfulness, cognition, workplace, organizations

## Abstract

Work stress is consistently linked with the deterioration of cognitive and mental health, limitations in everyday workplace performance, and an increased risk of developing diseases. A common thread binding these consequences appears to be stress-associated alterations in neuropsychological functions and affective domains, especially those reliant on hippocampal, prefrontal, and amygdala brain area. Although research broadly supports the claim that the practice of mindfulness meditation for the reduction of the consequences of stress and the promotion of health exert positive effects on workplaces, the precise neuropsychological benefits of Mindfulness-based interventions (MBIs) in the context of organizations remain elusive. In this review, we will analyze the impairments imposed by stress on the brain areas and functions and the benefits of MBIs from a neuropsychological point of view. This is significant since there is a centrality of cognitive functions in core processes necessary for work achievements, such as emotion regulation, problem-solving, and learning. The promotion of wellbeing is a responsibility shared between workers and organizations. Developing healthy environments allows workers to exercise greater control over their work, face work challenges, work productively and develop their talent.

## Introduction

The economy and the world of work have been transformed by globalization and technological innovation. In recent years, the need to adopt new types of jobs, an increase in demands, increased pressure of work quality and productivity, and increased time restrictions have been observed systematically (Spontón et al., 2019). Phenomena relating to work stress have increased rapidly as a result, which represents a major health risk factor (Morera et al., 2019).

It is therefore imperative to develop a variety of strategies to protect workers’ health (Vella and McIver, 2018). Within these strategies, initiatives have been carried out for years, such as the promotion of greater personal control, looking after democratic participation and a sense of community among workers through unions (Hagedorn et al., 2016), and job design by the employer with the promotion of “salutogenic” environments (Jenny et al., 2017). In the attempt to improve these work environments, mindfulness appears as a contemporary intervention with promising results in different settings and populations (Ludwig and Kabat-Zinn, 2008; Bohlmeijer et al., 2010).

There is an increasing body of evidence on the effectiveness of mindfulness-based interventions (MBIs) in improving health, wellbeing, and job satisfaction and in reducing workplace stress (Kersemaekers et al., 2018; Rupprecht et al., 2018, 2019). However, it is very important for organizations to contemplate a neuropsychological point of view since cognitive symptoms constitute an important cause of everyday performance limitations in the workplace and may be pre-clinical markers of the onset of ill-health (Sareen et al., 2006; Delphin-Combe et al., 2016; Fung et al., 2018). Integrated models are therefore necessary to account for how MBIs modify dysregulated emotional and neuropsychological responses to stress in organizations combining different benefits. This mini-review will analyze the impairments stress imposes on brain areas and functions and how MBIs can help from a neuropsychological standpoint.

### Neuropsychology of Stress

**Figure 1:**
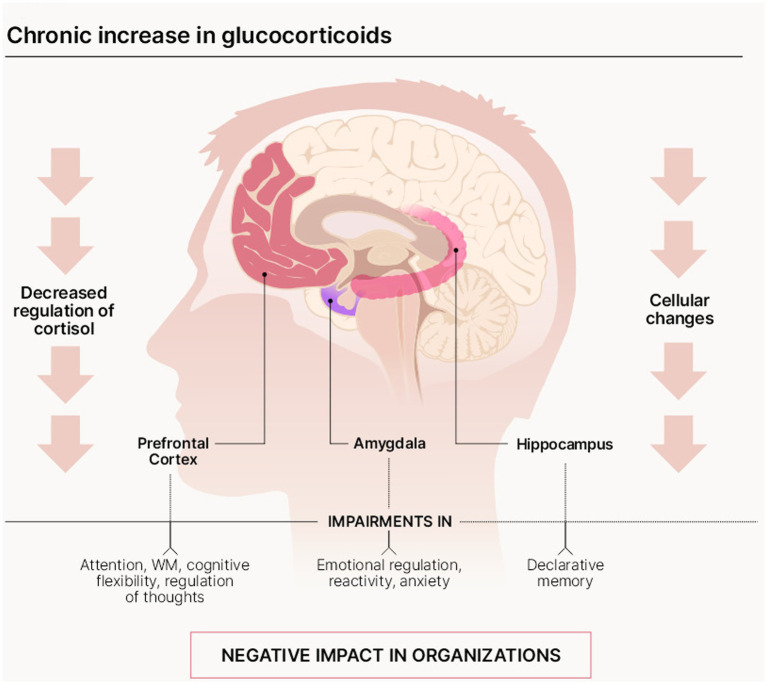
Impact of stress on main brain areas, cognitive functions, and affective domains.

When faced with a stressor, a person’s organism is biologically wired to activate a protective physiological response triggered by the HPA axis (interaction between the pituitary gland, the hypothalamus, and the adrenal glands), which produces glucocorticoid hormones, particularly cortisol (McEwen and Gianaros, 2011). Glucocorticoids (GC) affect the organism in multiple ways to increase its energy reserves and ensure adequate protection from, and adaptation to, the new environmental demands (Sapolsky, 2015; Cameron and Schoenfeld, 2018). When such stressful situations become repetitive or long-lasting, the organism’s reaction is to produce these chemicals in excess (McEwen and Wingfield, 2010), or to manage them inefficiently. This affects several regions of the cognitive and affective domains (Hermans et al., 2014). There are clearly serious effects of stress on the structure of neurons in the prefrontal cortex, the hippocampus, and the amygdala (Figure 1).

### Impact of Stress on the Prefrontal Brain

Experimental data support the notion that stress has a harmful effect on the higher order cognitive functions that depend on the prefrontal cortex (PFC), mainly working memory and cognitive flexibility processes (Arnsten, 2009; Mika et al., 2012; McEwen and Morrison, 2013). This area of the brain has been found to contain moderate or high concentrations of glucocorticoid receptors (Kuipers et al., 2003; Kavushansky and Richter-Levin, 2006). According to different studies, the PFC is the region of the brain that responds to stress with the highest sensitivity; quick changes have been observed in the functions depending on it (Brown et al., 2005). Moreover, research on burnout patients has shown that impairments related mainly to the speed, attention and executive function of cognitive functions persist for 3 years or more even after the stressor disappears (Jonsdottir et al., 2017).

Data of this sort are essential for organizations, as the PFC is the brain region responsible for executing complex cognitive processes. Working memory (WM) for example, which is dependent on the PFC, involves the ability to consider a very recent event, or to retrieve particular information stored in long-term memory, and to make use of this representational knowledge in the present, thus regulating behavior (Arnsten, 2009). Thanks to WM, goal-relevant information can be maintained and manipulated selectively over short intervals (ranging between a few seconds and several minutes), with no distraction from irrelevant information. WM has a limited capacity and is necessary for complex thought processes and fluid behavior. It is crucial to reasoning, planning, and decision-making (Jha et al., 2019).

The PFC is also involved in the processes that regulate our thoughts, actions, and emotions by protecting mental representations from being interfered by external or internal distractions. It is crucial to inhibiting inappropriate behaviors and promoting operations relevant to tasks at work and everyday life activities. The operations governed by the PFC enable behavior to be flexibly regulated and help us to produce appropriate responses to a changing environment (Arnsten, 2009). One example of this is the ability to shift attentional focus, to alter decision-making as it takes place, and to modify contingencies.

### Impact of Stress on the Hippocampal Brain Area

When we are chronically exposed to high levels of glucocorticoids, we suffer electro-physiological alterations that generate dysfunction, atrophy, and death of neurons in areas of the brain like the hippocampus that are essential to learning and memory (McEwen et al., 2016). Studies with both rodents and primates (Sandi, 2013) have shown that exposure to chronic stress results in atrophy of the dendritic prolongations of the main neurons of the hippocampus, reducing the interneuronal synaptic connections and, hence, neural and synaptic plasticity (Kim et al., 2015).

In the workplace, impairment of these functions is likely to weaken an individual’s ability to process information in new situations, for instance, and to make decisions on how to cope with incoming stimuli or stressors (Gianaros et al., 2007; Kim et al., 2015). It also weakens memory and learning processes, which are indispensable for achievements in the working environment.

### Stress and Amygdala

The amygdala enhances the secretion of glucocorticoid in response to stress and suffers stress-induced changes in neuron cytoarchitecture (Hölzel et al., 2010). Unlike the prefrontal cortex and the hippocampus, dendritic arborizations and spines of neurons in the amygdala are increased by stress. It has been proposed that these changes are the mechanisms that underlie stress-associated anxiety disorder, among others.

One of the amygdala’s functions in processing stressors is to rapidly assign emotional significance to events in the environment and is therefore believed to be involved in coordinating the behavioral changes induced by stressors, especially when faced with adverse environmental conditions and stressors that have a negative effect on health (Gianaros et al., 2007; McEwen and Gianaros, 2010; McEwen and Wingfield, 2010).

## The Impact of MBIs: A Neuropsychological Point of View

**Figure 2:**
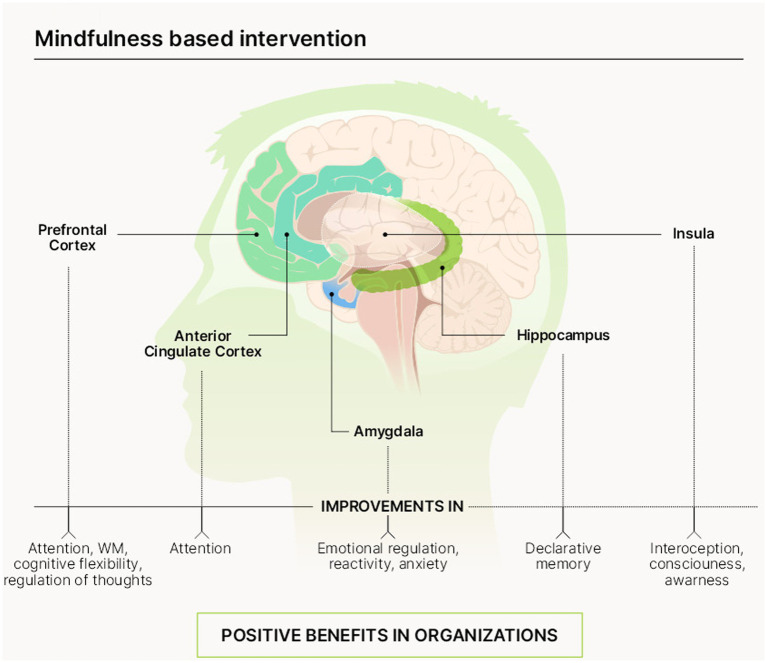
Impact of MBIs on main brain areas, cognitive functions, and affective domains.

Mindfulness is a mental practice that has been described as a “systematic form of mental training” which impacts on multiple affective domains and cognitive functions (Gill et al., 2020). The following sections will be devoted to describing the main findings on how MBIs influence affective domains and cognitive processes, and the areas in the brain responsible for these functions (Figure 2).

### Influence of Mindfulness on Attention and Executive Functions

Reviews generally associate mindfulness with attention improvement (Chiesa and Serretti, 2011; Tang et al., 2015). From a theoretical viewpoint, attention is generally regarded as a central aspect of mindfulness that has an impact on various characteristics as a neuropsychological construct (Hölzel et al., 2011; Vago and Silbersweig, 2012). For instance, moment-to-moment application, and sustainment and redirection (in the event of distraction) of attention to a target object are typically involved in mindfulness meditation (Tang et al., 2016). Trait-like changes in attentional brain networks have been observed to result from consistent practice over time; these networks apparently constitute a neural mechanism through which the abilities developed during training transfer into everyday life (Hasenkamp and Barsalou, 2012; Hasenkamp et al., 2012).

To be more specific, three distinct but interrelated networks are often attributed to attention (Posner, 2012): (i) alerting, defined as preparing for an impending stimulus; (ii) orienting, defined as targeting specific sensory input selectively based on modality or location; and (iii) executive attention (conflict monitoring), defined as monitoring task-relevant stimuli in a goal-directed manner while surrounded by competing task-irrelevant stimuli.

Short-term training studies have not shown significant effects on alerting (Jha et al., 2015) while intensive meditative experience and long-term mindfulness training do seem to improve alerting (MacLean et al., 2010). On the other hand, improvements in orienting have been reported after short- and long-term training studies (Jha et al., 2007; MacLean et al., 2010). Studies concerning the relationship between mindfulness and executive attention have shown that there was a relationship between higher trait mindfulness and lower interference in accuracy and reaction time, in keeping with enhanced executive attention (Smart and Segalowitz, 2017; Lin et al., 2019). However, these findings are as yet inconclusive and require further study (MacCoon et al., 2014).

What is more, there is increasing evidence that attentional processes play a critical role in supporting successful working memory (WM), one of the PFC’s main functions (Gazzaley and Nobre, 2012). Encoding requires selective attention to focus. Reflective attentional resources must “refresh” memory traces over the maintenance interval for encoded information to be successfully maintained (Shipstead et al., 2012; Camos et al., 2019). Refreshing involves enhancing, prolonging, and strengthening the representations of task-relevant data being held in WM. Memory traces may become unstable without refreshing, which leads to WM failures. Maintaining WM also requires attentional disengagement to ensure that distracting or obsolete information is removed from capacity-limited WM storage as it is no longer task-relevant (Shipstead et al., 2016; Lewis-Peacock et al., 2018).

The effects of meditation in brain areas related to attentional control and executive attention have also been found in a number of neuroimaging studies. One of the brain regions most widely mentioned in relation to these effects is the anterior cingulate cortex (ACC). This region contributes to executive attentional functions by detecting a cognitive conflict and alerting the dorsolateral prefrontal cortex (DLPFC), a top-down-regulated system, to resolve the conflict (Sridharan et al., 2008). Earlier studies have suggested that the stages of meditation practice—early, intermediate, and advanced—affect the change in brain activation levels and showed a quadratic relationship (Tang et al., 2015). A different amount of effort is required at each stage to exercise self-regulation, inducing different levels of activation in the brain. The participant needs to exert effortful control to stay engaged in the early stage of meditation, with increased activation in lateral PFC and the ACC (Tomasino and Fabbro, 2016). Next, somewhat less effort is required in the intermediate stage to reduce mind-wandering, while little or no effort is needed in the advanced stage. Participants with higher levels of meditation expertise therefore show less activation in the brain areas that had shown increased activation in the early stage (Kwak et al., 2020).

Moreover, as a consequence of the increase in the dorsolateral prefrontal cortex, there is a reorganization of the networks in which it is involved, among which the frontolimbic network, which mediates emotional regulation, stands out. One of the physiological mechanisms that supports the hypothesis that the practice of mindfulness improves attention function is based on the increase in α oscillations, a neuronal rhythm in which cells emit action potentials at a frequency of about 8–12 shots per second (Diez and Castellanos, 2022). As Jensen and Mazaheri (2010) emphasize, α oscillations do not represent a brain state or a state of relaxation, but rather a specific inhibition that prevents internal interference and enhances top-down control mechanisms, which are key to the execution of a task requiring focused attention. The α oscillations therefore reflect the priority of the information, inhibiting the areas with irrelevant tasks at that moment.

For organizations, an important aspect concerns how MBI exercises can train workers’ attention and WM processes. Cognitive training perspectives suggest that cognitive processes engaged by a particular activity can be strengthened by engaging repeatedly in the activity (Clare et al., 2003; Simons et al., 2016). In line with this research, the content of the formal exercises typical of MBI programs takes on greater relevance. Among these exercises, we find focused attention (FA), open monitoring (OM), yoga movements, and body scanned exercises (Lutz et al., 2015). While doing them, the individual wilfully decides that for an uninterrupted period of time they will fix their attention on the mindful exercise. In this way, the exercises require the individual to maintain the task set in WM, as the specific instructions to be engaged in for the practice period are held in mind (Lutz et al., 2015). The different studies conducted to investigate this hypothesis have yielded positive results (Black et al., 2011; Quach et al., 2016; Course-Choi et al., 2017).

Although the impact of mindfulness on attention and executive functions (as described above) has been analyzed by numerous researchers, there is as yet no conclusive evidence. Lao et al. (2016), for example, focused on 18 controlled trials of Mindfulness Based Cognitive Therapy and Stress Reduction programs (MBCT and MBSR) and were unable to find any significant evidence of improvement in attention. All that was found is preliminary evidence of improvement in working memory, cognitive flexibility, and meta-awareness. Because these cognitive functions are important in workplaces and organizations, MBIs in these cognitive functions needs considerable research in order to analyze the benefits for different work settings.

### Influence of Mindfulness on Memory and Learning Processes

Recent work with mindfulness indicates that its practice can positively affect verbal learning and memory. Increased attention may be a potential explanation for this improvements. Mindfulness may have an impact on autobiographical memory by training individuals to be attentive to particular aspects of their experiences, promoting greater accuracy in encoding and retrieval, which improves specific memory (Heeren et al., 2009). Other studies have proposed that improvements in verbal learning and memory may result from mindfulness *via* its effect on the encoding process of learning rather than altering attention (Lueke and Lueke, 2019). For example, some studies showed that mindfulness can help to reduce mind-wandering (Killingsworth and Gilbert, 2010) and proactive interference (Greenberg et al., 2019). Although mindfulness may help free up cognitive resources required for peak verbal learning and memory performance that would otherwise be engaged in task-unrelated thoughts, the evidence and findings, though as yet preliminary, are promising (Lao et al., 2016).

Neuroimaging studies have provided researchers with more data on this issue, as there is documented evidence of the impact of mindfulness training on the structure of the hippocampus. Magnetic resonance imaging (MRI) studies of 8-week mindfulness training programs have shown increases in the density of hippocampal grey matter in healthy individuals (Hölzel et al., 2011) as well as a reduction in hippocampal atrophy in adults with mild cognitive impairment (Wells et al., 2013) after training. Cross-sectional studies support these results after mindfulness training, indicating greater hippocampal grey matter among long-term meditators in comparison with non-meditators (Luders et al., 2013, 2015).

### The Impacts of Mindfulness on Perceived Stress and Emotional Regulation

It has been shown that mindfulness meditation facilitates emotional regulation and attention self-regulation (Kabat-Zinn, 2019). Earlier studies (Arredondo et al., 2017; Heckenberg et al., 2018) had pointed out how implementing the practice of mindfulness can reduce harmful physiological effects on health by acting on the conscious evaluation of stimuli and by grading the reaction to them, since these stimuli would not automatically be considered a threat. Along similar lines, Feldman et al. (2007) found that there was a strong correlation between personal mindfulness reports and self-reported use of adaptive emotional regulation strategies. MBIs are also effective in increasing high and low diurnal cortisol slopes, which suggests an improvement in HPA axis regulation (Heckenberg et al., 2018).

Nevertheless, there is still debate as to the exact relationship between mindfulness and emotional regulation. Some authors claim that consciousness without judgment, typical of mindfulness practices, facilitates a healthy relationship with emotions (Hayes and Feldman, 2004), making it possible for people to fully experience and express their emotions (Bridges et al., 2004), helping to reduce mechanisms like avoidance, suppression or over identification with emotions, which produce anxiety or rumination.

These data are supplemented by neuroimaging studies. Kral et al. (2018), for example, evaluated the impact of training in long- and short-term mindfulness meditation on how the amygdala responds to emotional pictures in a healthy, non-clinical adult population using functional magnetic resonance imaging. The authors conclude that affective responding can be improved by meditation training through heightened amygdala–PFC connectivity and reduced amygdala reactivity, as this has a salutary effect on the ability to regulate emotions (Banks et al., 2007; Lee et al., 2017).

The impact of MBIs on emotional regulation is crucial for organizations and, according to the literature, mindfulness interventions are a useful resource that favor improvements in employee health. For example, previous studies claim that mindfulness can raise awareness of early warning signs of stress prior to it becoming unmanageable and that it also provides tools to offset the negative effects of stress on wellbeing and job performance (Christopher et al., 2018). Other authors have put their focus on the resilience building qualities that can protect workers from adverse outcomes and help them develop in a complex and rapidly changing work environment (Kinman et al., 2020).

### Other Key Brain Areas and Mechanism in MBI: Interoception and Insula

Another essential point in MBI research is the relationship between interoceptive awareness and mindfulness and the insula, its neurobiological brain area (Sharp et al., 2018; Gibson, 2019). Recent studies indicate that interoception is crucial to mindfulness and that some of its benefits are better described as resulting from changes in neuroplasticity in the insula (Sharp et al., 2018) and the neural circuits surrounding it, which can promote dispositional conscious attention (Gibson, 2019).

Interoceptive learning is believed to be a complex process that can be accomplished using MBI (Gotink et al., 2016). Most meditation traditions are known to incorporate attention to internal body sensations as part of the practice, especially in the early stages of the learning process. The body sensations most commonly focused on are the breath, the position of the joints (proprioception), the degree of muscle tension, and heartbeat. It has been suggested that attending to interoceptive sensations can result in improved awareness of these sensations, and that practizing meditation leads to enhanced awareness of a number of other internal events, as among them the ongoing experience of thoughts and emotions (Allen et al., 2012).

## Discussion

The purpose of this mini-review is to examine what cognitive abilities and psychological variables are enhanced with MBIs and their association with those impaired by chronic stress, focusing mainly on how organizations benefit from MBIs. Unexpectedly, existing studies that investigate the neuropsychological impact of MBIs on cognitive functions of samples taken from such work environments were inconsistent. Nevertheless, those concerning the impact stress has on cognition and the benefits of MBIs in employers’ and employees’ brain functions are undoubtedly promising.

Mindfulness has grown considerably in popularity, but so have misconceptions regarding what it is and how both the mind and the brain are affected. As Wielgosz et al. (2019) points out, the popularity of the MBI has generated increasingly ambitious claims about its clinical benefits that are not always supported by scientific data, generating inflated expectations and leading to this type of intervention being prematurely deployed in contexts where it has not been sufficiently tested.

Moreover, despite the fact that most mindfulness training derives from the original MBI model (Kabat-Zinn, 1994), key variations, such as duration of practice (total engagement time) and intensity (hours per day), are often overlooked. This leads to inappropriate comparisons and non-generalizable conclusions. For this reason, and for MBIs to be considered credible and evidence-based interventions, we first suggest conceptualizing MBIs not as a particular technique, but as a family of practices targeting multiple neurobehavioral systems involved in psychopathology. This family of practices should be linked to clear standards (form of implementation, required training, fidelity, and cross-cultural adaptation) that guarantee the safety and efficacy of the intervention. For this, it is important to know precisely which mechanisms are involved in MBI.

It is important to communicate new findings from contemplative neuroscience about the brain mechanisms and mental processes of mindfulness practices with corresponding modesty (Van Dam et al., 2018). A deeper understanding of what the effects of the MBI are, the mechanisms involved, and the circumstances in which it can be applied will make it possible to determine more clearly the implementation rules that will guarantee the desired effects.

It is necessary for future studies to continue to examine the psychological and neurocognitive benefits of MBIs in the workplace. For example, from a neuropsychological perspective, how is it that mindfulness impacts on different functions and not just the trained cognitive function? Although a greater understanding of the mechanisms involved is required, we can affirm that MBI is considered to be an efficient, effective, and low-cost resource for the reduction of work stress, making it possible to model employers’ and employees’ performance (Jamieson and Tuckey, 2017) and thus improve their main work abilities, cognitive functions, and mental health, leading to significant benefits for organizations (Arredondo et al., 2017).

## Author Contributions

FG-P and LM conceived of the presented idea and discussed the results and contributed to the final manuscript. FG-P developed the theory and wrote the manuscript with support from LM. LM supervised the findings of this work. All authors contributed to the article and approved the submitted version.

## Funding

This work was supported by the Universidad Siglo 21, Argentina.

## Conflict of Interest

The authors declare that the research was conducted in the absence of any commercial or financial relationships that could be construed as a potential conflict of interest.

## Publisher’s Note

All claims expressed in this article are solely those of the authors and do not necessarily represent those of their affiliated organizations, or those of the publisher, the editors and the reviewers. Any product that may be evaluated in this article, or claim that may be made by its manufacturer, is not guaranteed or endorsed by the publisher.
